# Ultrafast photooxidation of semireduced flavin in fatty acid photodecarboxylase

**DOI:** 10.1126/sciadv.adz1904

**Published:** 2025-09-19

**Authors:** Marten H. Vos, Elsa Balduzzi, Damien Sorigué, Alexey Aleksandrov

**Affiliations:** ^1^ Laboratoire d’Optique et Biosciences, CNRS, INSERM, Ecole Polytechnique, Institut Polytechnique de Paris, 91120 Palaiseau, France.; ^2^ Institute of Biosciences and Biotechnologies, BIAM Cadarache, Aix-Marseille University, CEA, CNRS, 13108 Saint-Paul-lez-Durance, France.

## Abstract

The initial photoproduct of the natural photoenzyme fatty acid photodecarboxylase involves the flavin anion radical flavin adenine dinucleotide (FAD^•–^). Using spectrally resolved ultrafast transient absorption spectroscopy, we demonstrate that FAD^•–^ photoexcitation in the absence of substrate leads to the formation of the oxidized flavin FAD_ox_ (the resting state in the catalytic cycle) within 100 femtoseconds. While this feature is similar to that occurring in flavoprotein oxidases, the ensuing photocycle is more complex. Upon excitation at the lowest-energy transition, the ejected electron is initially captured as a hydrated electron (_e_^–^_H_) before transferring to a secondary acceptor in 2.5 picoseconds and returning to the flavin in 37 picoseconds. This implies that _e_^–^_H_ can be generated within a protein environment, an unprecedented finding. This assessment is supported by molecular dynamics simulations showing an expansion of the flavin-binding pocket without substrate, allowing water molecules to fill the void. Our results may pave the way to developing unconventional photocatalytic processes.

## Teaser

A hydrated electron was photogenerated within a protein upon excitation of a reaction intermediate of a natural photoenzyme.

## INTRODUCTION

Flavins are colored cofactors in diverse classes of proteins that display highly versatile redox properties. They are exploited by nature, as electron and/or proton storage intermediates in many enzymatic reactions with substrates, due to their ability to adopt different oxidation (oxidized, one- and two-electron reduced) and protonation states (*1*). Upon population of excited states by absorption of light, flavins can also undergo redox reactions with neighboring molecular entities (either amino acids or substrate molecules) that are otherwise thermodynamically impossible from the ground state (*2*). In some flavoproteins, these photoreactions play a functional role, as seen in blue-light photoreceptors and in the rare flavin-based photoenzymes (*3*). However, in most flavoenzymes, photoreactions do not appear to contribute directly to catalysis.

The most studied flavin-involving photoreactions are those initiated by exciting the most stable redox forms, i.e., oxidized or fully reduced. Photoreduction of oxidized flavins plays a functional role in photocatalysis by fatty acid photodecarboxylase (FAP) (*4*–*7*) and in most blue-light–sensing photoreceptors (*8*). In other flavoproteins, this process may serve a protective function by preventing the formation of singlet oxygen through triplet excited states (*9*, *10*). Photooxidation of fully reduced flavins plays a functional role in photocatalysis by DNA photolyases (*11*) and in several engineered biophotocatalysts (*12*). Flavins can also adopt semireduced radical states, which are often functionally important as intermediates but typically difficult to stabilize. A notable exception is DNA photolyase, where the neutral semireduced radical state of the flavin adenine dinucleotide (FAD) cofactor FADH^•^ remains stable even under aerobic conditions and can be converted, by photoreduction involving nearby tryptophan residues (*13*), into the catalytically active fully reduced form.

Anionic flavin radicals have been shown to be stabilizable under anaerobic conditions, only in a limited number of flavoenzymes. In particular, this is observed in flavoprotein oxidases, which harbor close by positively charged residues to stabilize superoxide catalytic intermediates (*14*), but which can also stabilize the flavin in the FAD^•–^ state (*15*). We have recently demonstrated that photoexcitation of this state [FAD^•–^ or, in the case of flavin mononucleotide (FMN), FMN^•–^] in a variety of these proteins invariably leads to very rapid (<100 fs) formation of the oxidized FAD_ox_ or FMN_ox_ state, followed by recombination to the radical anion state in 10 to 20 ps (*15*). It was made plausible that the cationic (histidine or arginine) residues nearby act as electron acceptors in this case and are thus converted to their neutral radical forms. This reaction cycle can thus be viewed as a light-induced combination of a charge pair, followed by a reseparation. By exploring ways to stabilize the initial photoproduct, the discovery of this photoreaction may eventually lead to a hitherto unknown class of photobiocatalytic reactions.

Among the flavoprotein oxidases investigated are glucose oxidase (GOX), an enzyme extensively used in biotechnological processes, and choline oxidase (*15*). Both enzymes, along with FAP, are members of the glucose-methanol-choline (GMC) family of oxidoreductases (*6*, *16*). In FAP, FAD_ox_ is the catalytically active resting state, and FAD^•–^ is the catalytic intermediate state resulting from electron transfer (ET) from the fatty acid (FA) substrate to the excited flavin. This state is formed in 300 ps and decays in 100 ns by a reoxidation reaction involving a closeby essential arginine residue (acting as a proton donor) and the decarboxylated substrate radical (*5*, *6*). A major result of a recent paper on the optical spectroscopy of FAP by Zhong and coworkers (*17*) was that the FAD^•–^ state can be easily stabilized in high yield via anaerobic photoreduction. In subsequent work, the same group investigated the photoproducts of this state by single-wavelength transient fluorescence and absorption spectroscopy (*18*). Their results, interpreted in terms of complex excited-state dynamics involving multiple electronic states, led them to dismiss the occurrence of photooxidation of FAD^•–^ based on a lack of signal in the red spectral region, despite a substantial absorption increase in the blue region characteristic of FAD_ox_. Intrigued by these findings, we reinvestigated the FAD^•–^ photochemistry in FAP using fully spectrally resolved ultrafast optical spectroscopy. Our findings not only confirm generally complex reaction dynamics but also provide compelling evidence for the formation of FAD_ox_ in the FAD^•–^ photoreaction and strongly support ultrafast flavin anion radical photooxidation as a general excited-state deactivation pathway. Furthermore, evidence for the formation of a hydrated electron (e^−^_H_) in this process is presented, providing a unique example of electron hydration within a protein cavity.

## RESULTS

### Absorption spectra

**Fig. 1.**
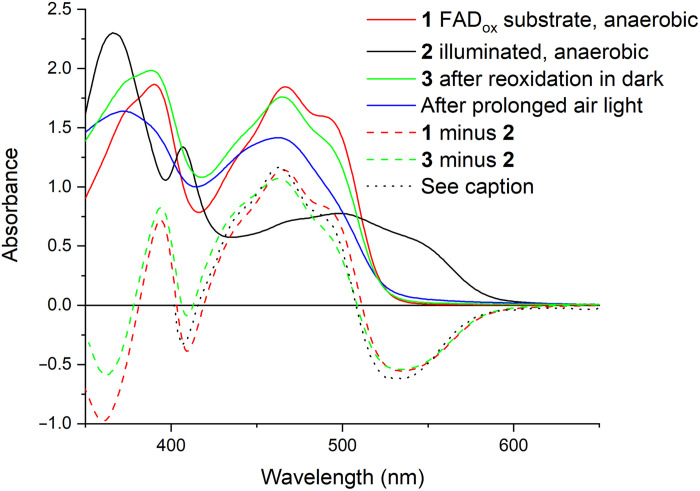
Absorption spectra. Solid lines: Steady-state absorption spectra of *Cv*FAP in the anaerobic FAD_ox_-substrate form (1; red), photoreduced FAD^•–^ form (2; black), FAD_ox_ form after reoxidation by air in the dark (3; green), and prolonged exposure to air in ambient light (4; blue). Dashed lines: Difference spectra 1 minus 2 (red) and 3 minus 2 (green). The black dotted line represents the normalized (FAD^•–^ minus FAD_ox_ substrate) spectrum obtained by time-resolved spectroscopy of the FAD_ox_-substrate complex and is taken from (*5*).

Figure 1 shows the steady-state ultraviolet-visible absorption spectra of different forms of FAP from *Chlorella variabilis* (*Cv*FAP). The initial FAD_ox_-substrate complex (red) kept in the dark displays marked vibrational structure and a highly red-shifted absorption, characteristic of FAP (*5*, *6*). From this complex, the semireduced FAD^•–^ form (black) is obtained upon anaerobic photoreduction, even in the absence of a reducing agent. The spectrum of this form is very similar to that obtained by Wu *et al.* (*18*) upon photoreduction in the presence of dithiothreitol. Upon reaerating the FAD^•–^ form in the dark, FAD_ox_ is reformed with somewhat different spectral properties (in particular, a lower shoulder at ~500 nm), presumably due to the absence of the substrate. Upon prolonged exposure to air and light, disappearance of the vibrational features and blue-shifting are observed, and a spectrum resembling that of FAD in solution remains, reflecting a photodegraded form of the enzyme.

Relevant corresponding difference spectra are also shown in Fig. 1. The steady-state difference spectrum of the FAD_ox_-substrate and FAD^•–^ forms is very similar to the inverse of that obtained for the transient absorbance difference in the catalytic photocycle after decay of the FAD_ox*_ excited state in 300 ps that was assigned to FAD^•–^ (*5*). The high similarity between these spectra also implies that the presence of the CO_2_ and FA radical initial photoproducts in the active site does not strongly influence the FAD^•–^ electronic spectrum.

### Photocycle under 560-nm excitation

**Fig. 2.**
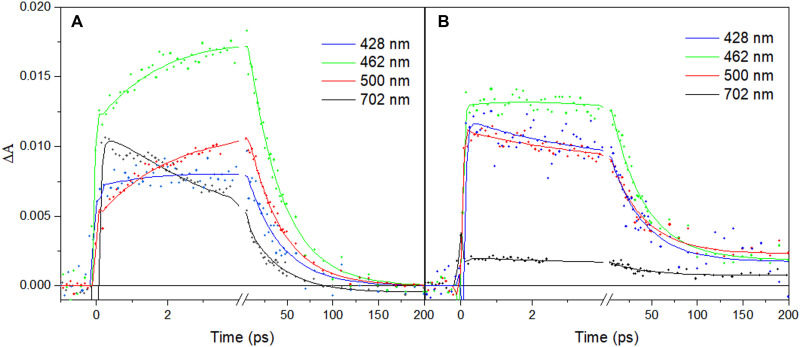
Transient absorption kinetics at selected wavelengths of *Cv*FAP in the FAD^•–^ form. Excitation at 560 nm (**A**) and 390 nm (**B**). The solid lines are from global fits as described in the text. Note the break in the time axes.

**Fig. 3.**
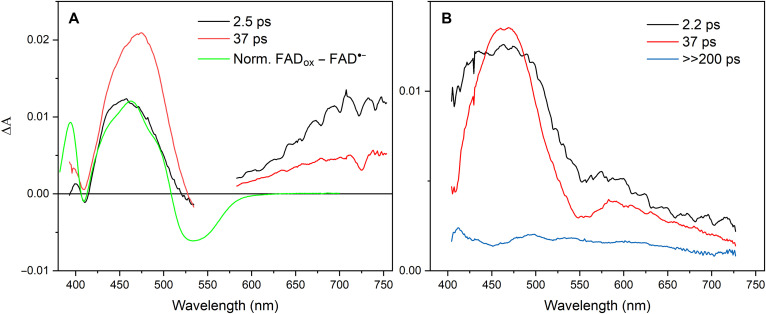
SAS of transient absorption experiments of *Cv*FAP in the FAD^•–^ form. Excitation at 560 nm (**A**) and 390 nm (**B**). The green line in (A) corresponds to spectrum 3 minus 2 in Fig. 1. Norm., Normalized.

We now turn to the photoproducts of the FAD^•–^ form. Figure 2A shows transient kinetics upon excitation at 560 nm, in the lowest FAD^•–^ transition (D_0_ → D_1_). These conditions also avoid excitation of any other flavin forms (FAD_ox_ or two-electron–reduced FAD forms). The strongest signal is observed at 462 nm, close to the maximum of the FAD_ox_ form. Here, the signal shows an instantaneous rise (<100 fs), followed by a slower rise in ~2.5 ps and a full decay in 37 ps (time constants from a global fit). The shape and time constants of the kinetics are very similar to the normalized kinetics reported for 550-nm excitation by Wu *et al.* (*18*). Our full spectral measurements (fig. S1) allow us to perform a global analysis of the ensemble of data. Figure 3A shows the corresponding species-associated spectra (SAS). We first concentrate on the blue side of the spectra. The initial SAS, corresponding to a species populated within our time resolution, displays a clear ~70-nm-wide induced absorption band centered near 460 nm. In ~2.5 ps, the band further increases by almost a factor of 2 and modestly changes in shape. Comparison with the steady-state FAD_ox_-minus-FAD^•–^ spectrum shows that both spectra are doubtlessly predominantly due to FAD_ox_ formation. These data thus indicate that photon absorption by FAD^•–^ leads to quasi-immediate FAD_ox_ formation [as in other flavoprotein oxidases (*15*)] in ~50% of the enzymes and in the remainder FAD^•–*^ excited-state formation, followed by delayed (2.5 ps) FAD_ox_ formation. In 37 ps, rereduction of the flavin to the initial FAD^•–^ form then occurs. Wu *et al.* (*18*) argued against the possibility of FAD_ox_ formation on the basis of the observed FAD^•–*^ fluorescence and the signals in the red part of the spectrum. We note that the reported FAD^•–*^ fluorescence decay in a few picoseconds supports our interpretation of partly delayed FAD_ox_ formation from FAD^•–*^.

**Fig. 4.**
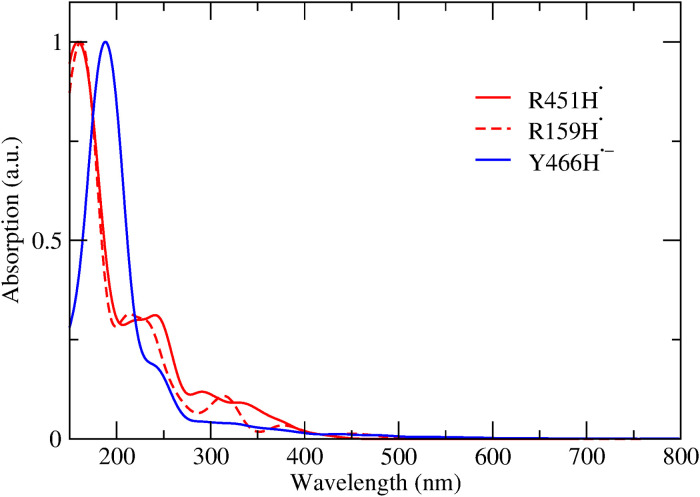
Simulated absorption spectra of amino acid radicals within the *Cv*FAP protein. R451, R159, and Y466 radicals are modeled in an explicit solvent environment. Each spectrum corresponds to the case in which the indicated residue acts as the electron acceptor in the ET reaction.

At the red side of the spectrum, a broad induced absorption, which reaches a spectral maximum at ~730 nm, is observed within 100 fs (Fig. 3A). This feature, with an amplitude similar to that of the initial FAD_ox_–induced absorption around 460 nm, is not expected based on the steady-state FAD_ox_-minus-FAD^•–^ spectrum. It also does not follow the kinetics of the FAD_ox_ population, as it decays in two phases of 2.5 and 37 ps. As stated above, this signal cannot be due to excitation of any residual FAD_ox_ because (a) FAD_ox_ does not absorb at 560 nm (Fig. 1) and (b) FAD_ox_* in *Cv*FAP has very different spectral properties (*5*). It could, in principle, be due to (a) the fraction of photoexcited FAD^•–^ that does not immediately form FAD_ox_ but instead occupies the excited-state FAD^•–^* or (b) the absorption of an electron acceptor. The first possibility appears unlikely in view of (i) the very strong initial induced absorption band and (ii) the observed multiphasic decay, which does not mirror the FAD^•–^* fluorescence decay that is completed in a few picoseconds (*18*). Concerning the second possibility, the cationic residues histidine (HisH_2_^+^) and arginine (ArgH^+^) have been proposed as potential electron acceptors within the protein moiety (*15*), yielding HisH_2_^•^ or ArgH^•^ as reduced neutral radical products, respectively. No histidine residues likely to be protonated are present near the isoalloxazine ring in either the substrate-bound crystal structure or our molecular dynamics (MD) simulations of the FAD^•–^ substrate–devoid system. In contrast, several arginine residues are positioned near the isoalloxazine ring in the structure. However, the absorption spectrum of the ArgH^•^ radical remains experimentally uncharacterized. To address this, we computed its spectrum using quantum chemical methods in the gas phase (fig. S1) and within the *Cv*FAP protein environment for the two residues closest to the flavin, R451 and R159 (Fig. 4). In both cases, ArgH^•^ is not predicted to absorb above 400 nm. These results allow us to also exclude reduced arginine as the source of the red or near-infrared absorption band experimentally observed.

Two additional residues located near the flavin—cysteine (C342) and tyrosine (Y466)—were evaluated for their potential involvement in ET. Similar to the arginine radical, the Tyr^•–^ radical does not exhibit notable absorption above 400 nm, both in vacuum (fig. S2) and within the protein environment (Fig. 4). For cysteine, ET—modeled in both vacuum and within *Cv*FAP (C342)—was found to induce cleavage of the C─S bond, resulting in a radical species that also lacks absorption beyond 400 nm.

We note, however, that the induced absorption band at >600 nm in Fig. 3A strongly resembles that of a hydrated electron (*19*–*21*). In addition, the amplitude of the initially formed red to near-infrared band is similar to that of the induced FAD_ox_ band, consistent with their extinction coefficients being in the same range (*20*, *22*). Therefore, we assign the initially formed state upon excitation of the *Cv*FAP FAD^•–^ state to [FAD_ox__e_^–^_H_], where _e_^–^_H_ stands for a hydrated electron–like species. The molecular origin of this species will be discussed below.

**Fig. 5.**
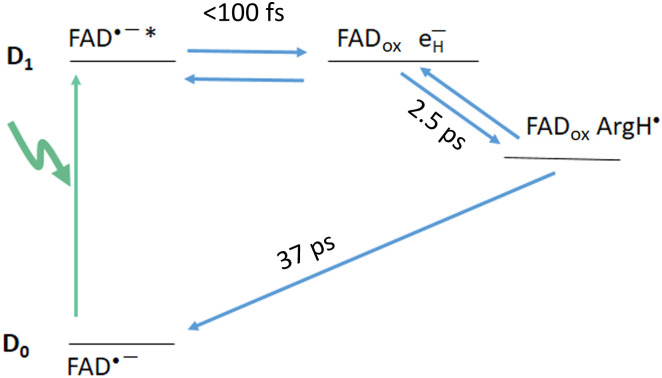
Schematic representation of the electron transfer process following excitation into the D_1_ excited state of FAD^•–^ in *Cv*FAP.

During the 2.5-ps phase, the FAD_ox_-induced absorption band in the blue rises further, whereas the _e_^–^_H_ band peaking near 730 nm decays. This indicates that during this phase, along with further oxidation of the flavin from the FAD^•–*^ state, the hydrated electron is partly transferred to a yet unknown electron acceptor X. During the 37-ps phase, the ensemble of thus-formed [FAD_ox__e_^–^_H_] and [FAD_ox_ X^−^] states reverses to the initial FAD^•–^ state. We propose a minimal reaction scheme that is in agreement with the ensemble of these observations and is depicted in Fig. 5. The D_1_ FAD^•–*^ excited state initially populated by excitation of the D_0_ FAD^•–^ ground state very rapidly (<100 fs) equilibrates with a state in which the electron is ejected by the flavin and hydrated by nearby water molecules, yielding [FAD_ox__e_^–^_H_]. In 2.5 ps, the _e_^–^_H_ state equilibrates with a lower-lying reduced state [FAD_ox_ X^−^], where we suggest X^−^ to be ArgH^•^, obtained by reducing ArgH^+^ (see above). This allows more FAD_ox_ to be formed and lowers the population of hydrated electrons. In this scheme, the first and second steps are reversible to account for the incomplete FAD_ox_ formation in the initial (<100 fs) step and for the incomplete _e_^–^_H_ depletion in the subsequent 2.5-ps step. Last, reversed ET to the FAD^•–^ ground state in 37 ps closes the photocycle.

### Photocycle under 390-nm excitation

As reported by Wu *et al.* (*18*), upon excitation predominantly into the D_2_ excited state of FAD^•–^ (here, 390 nm), a spectral response (Fig. 2B and fig. S3) is observed that substantially differs from that under excitation into the D_1_ excited state. However, our spectral analysis (Fig. 3B) shows that the initial response is dominated again by a strong induced absorption band around 460 nm, indicating that FAD_ox_ is formed in <100 fs as well. The kinetic evolution can be described with time constants of ~2.2 and 37 ps and also contains a long-lived (≫200 ps) phase. During the ~2.2-ps phase, the induced absorption around 460 nm, which is initially broader than that observed under 560-nm excitation, narrows rather than grows, indicating that a different process from that underlying the 2.5-ps phase under 560-nm excitation is at the origin of this phase. The spectral properties of this phase strongly suggest that FAD_ox_ is initially in a “hot” state due to the excess excitation energy and cools in a few picoseconds. The resulting band [37-ps Evolution-Associated Spectrum (EAS)] has a similar width to that observed under 560-nm excitation. Upon 560-nm excitation, the band disappears in 37 ps due to FAD^•–^ reformation.

**Fig. 6.**
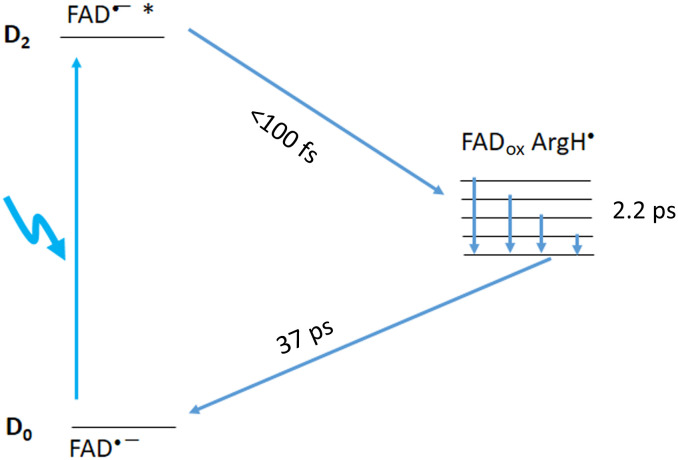
Scheme of electron transfer and cooling upon excitation into the D_2_ excited state of FAD^•–^ in *Cv*FAP.

Upon 390-nm excitation, the induced absorption at the red side of the spectrum is much lower and different in shape than that observed under 560-nm excitation (Fig. 3). This indicates that not much _e_^–^_H_ is formed under 390-nm excitation and presumably [FAD_ox_ ArgH^•^] is directly populated from the D_2_ FAD^•–^* state. We note that the shape of the EAS spectra above 600 nm is similar, including for the long-lived component, which is not present under 390-nm excitation. A plausible explanation of these spectra is that they arise from a fraction of two-electron–reduced flavin generated by the photoreduction process. These states only absorb below 550 nm and generally decay in a multiphasic way on the picosecond-nanosecond timescale (*23*–*25*). Together, our analysis indicates that upon excitation into the D_2_ state, [FAD_ox_ ArgH^•^] can be directly formed, and the resulting hot FAD_ox_ state relaxes in a few picoseconds. The reverse ET that repopulates the FAD^•^ ground state occurs in 37 ps. This is the same time constant observed following excitation into the D1 state (Fig. 6), supporting the assignment of a common final electron acceptor under both D1 and D2 excitations.

### Transient absorption upon FAD^•–^ excitation in GOX

**Fig. 7.**
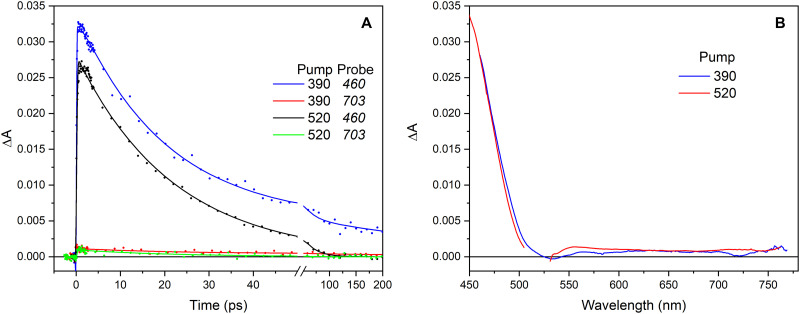
Transient absorption experiments of GOX in the FAD^•–^ form. (**A**) Kinetics at selected wavelengths. (**B**) Decay-associated spectra of the ~20-ps component observed under excitation at 520 nm (red) and 390 nm (blue). The data in both panels are normalized on the amplitude of the ~20-ps component at 460 nm.

In our previous work on FAD^•–^ and FMN^•–^ photoproducts in flavoprotein oxidases, the spectral evolution on the red side of the spectrum was investigated only under 390-nm excitation, and no _e_^–^_H_-like spectral features were observed (*15*). As these states were only observed under excitation into the D_1_ FAD^•–^ state of *Cv*FAP, to investigate whether these states are formed in other flavoproteins of the GMC family of oxidases, we now extended our investigations on GOX to the red spectral range with excitation into the D_1_ FAD^•–^ state. Figure 7 compares the results for excitation in the D_1_ state (520 nm for GOX) and the D_2_ state (390 nm). The results for 390-nm excitation are very similar to those reported previously, covering the full spectral range (*15*). They consist of a rise in <100 fs with a maximum at ~450 nm assigned to FAD_ox_ formation and a subsequent decay in ~20 ps assigned to the reformation of the FAD^•–^ state. The minor longer-lived phase observed only upon 390-nm excitation was assigned to trace amounts of FADH^•–^ in the sample (*15*). Notably, upon 390-nm excitation, only a very small signal is observed at the red side of the FAD_ox_ band, and the 20-ps Decay-Associated Spectrum (DAS) can be assigned to the [FAD_ox_ HisH_2_^•^] state (*15*). Upon 520-nm excitation, a very similar signal is observed in the blue-side FAD_ox_ absorption region, as reported before (*15*). Our present results also show that only a very weak broad signal is observed at the red side upon 520-nm excitation. The shape, intensity, and temporal evolution of this signal correspond well with those of the 20-ps DAS under 390-nm excitation. Therefore, in contrast to FAP, no indication of a sizeable signal assignable to an _e_^–^_H_-like species is observed upon FAD^•–^ D_1_ excitation in GOX.

### MD simulations

This work provides compelling experimental evidence that an electron ejected from the photoexcited flavin in *Cv*FAP can be captured by nearby water molecules, forming a hydrated electron–like species. This finding indicates that multiple interacting water molecules are present near the isoalloxazine chromophore in the FAD^•–^ state, in the absence of a bound FA. Crystallographic information is only available for the initial FAD_ox_ substrate–bound state and intermediate states, where the product molecules remain present in the protein (*5*). In the resting state, only three, not directly interacting, crystalline water molecules are found in the active site (*5*). However, in the present solution-phase experiments, the enzyme differs from the crystallographic states in two key ways due to the initial photoreduction procedure: (i) the active site is devoid of both substrates and products, and (ii) FAD is present in its reduced anionic radical form (FAD^•–^). To better understand the flavin environment in this state, we performed classical MD simulations.

**Fig. 8.**
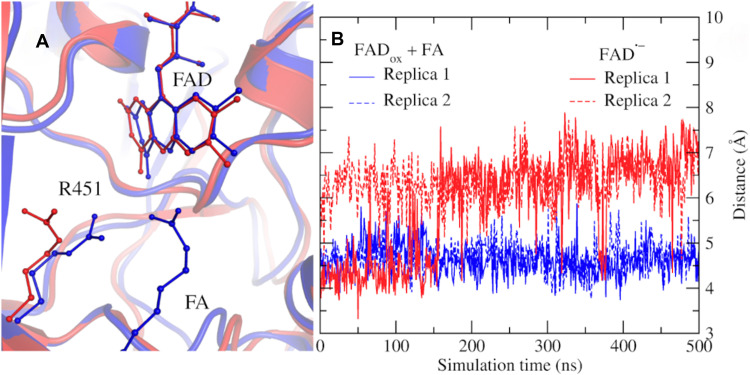
MD simulations of *Cv*FAP in different redox forms. (**A**) Structural superposition of average conformations from 500-ns MD simulations with FAD in its oxidized state bound to two FA molecules (blue) and in its reduced radical state without FAs (red). (**B**) Evolution of the distance between the isoalloxazine ring of FAD and the guanidinium group of R451 in two independent 500-ns simulations for each redox form.

Figure 8A shows a structural overlay of the average conformations obtained from MD simulations of *Cv*FAP with FAD in its reduced radical state (FAD^•–^, without FA) and in its oxidized state (FAD_ox_, with two FA molecules). The protein backbone and flavin conformations display minimal global differences between the two states. Notably, the isoalloxazine ring of FAD remains bent in both cases, with the butterfly bending angle (dihedral C_4_─N_5_─N_10_─C_9_) at 12.5° for FAD_ox_ (with 2FA) and 16.7° for FAD^•–^ (substrate-free), indicating that this structural feature is not induced by substrate binding (*5*). However, a pronounced difference was observed in the position of residue R451. In the substrate-devoid FAD^•–^ state, R451 frequently undergoes marked changes between two conformations on the hundreds of nanosecond timescale, resulting in the predominant population of a configuration with an increased distance between its guanidinium group and the isoalloxazine ring, as illustrated in Fig. 8B.

In simulations of the system containing FAD_ox_ and two substrate molecules, the distance between the isoalloxazine ring of FAD and the guanidinium group of R451 remained stable at ~4.5 Å, consistent with the crystal structure value of 4.2 Å. Throughout these simulations, the carboxylate oxygen atoms of the C_18_ FA maintained close interactions with R451, helping to stabilize its position. In contrast, simulations with reduced FAD (FAD^•–^) and no bound substrates revealed a notable increase in this distance, rising from ~4.2 to ~6.5 Å during the MD simulations. This conformational shift was consistently observed in two independent 500-ns simulations for each system, initiated from distinct starting structures (Fig. 8B). In both cases, the transition occurred either during the equilibration phase or within the first 50 to 100 ns of the production run. Short-lived fluctuations were also observed, during which R451 briefly approached the flavin, but these events were transient and typically resolved within about 1 ns, with R451 returning to its more distant orientation.

**Fig. 9.**
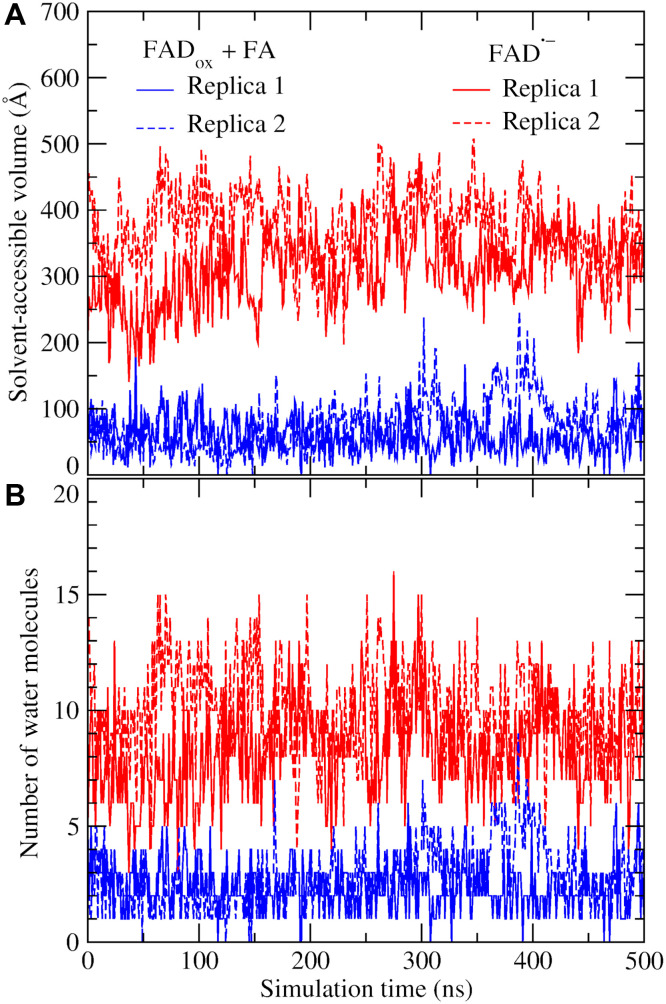
Water molecules in the active site. (**A**) Solvent-accessible volume of the pocket surrounding the N_5_ atom of FAD over the course of MD simulations. (**B**) Number of water molecules residing within the solvent-accessible region near the isoalloxazine ring of FAD.

To assess whether the increased distance between R451 and FAD is associated with enhanced solvent accessibility, we analyzed both the solvent-accessible volume of the pocket surrounding FAD (fig. S4) and the number of water molecules within this pocket over the course of the MD simulations (Fig. 9). In the system containing FAD_ox_ and two FAs, the volume around the N_5_ atom of the isoalloxazine ring remained stable at ~50 Å^3^ (blue trace), with an average of two to three water molecules present. In contrast, for the reduced FAD^•–^ system devoid of substrates, the pocket volume expanded to ~300 Å^3^ (red and black traces; Fig. 9A), accommodating up to eight water molecules (Fig. 9B).

In the FAD^•–^ complex devoid of FAs, the region near FAD previously occupied by the carboxyl group of the C_18_ FA becomes filled with water molecules (Fig. 9B). Furthermore, the conformational change of R451 (Fig. 8A) facilitates the entry of additional water molecules into the pocket, increasing solvation of both the ionized R451 and the flavin. This is further corroborated by radial distribution function (RDF) analysis (fig. S5), which reveals a closer peak for water around the N_5_ atom of FAD in the reduced, FA-free state (~2.8 Å) compared to the oxidized state with two FAs (~3.3 Å).

**Fig. 10.**
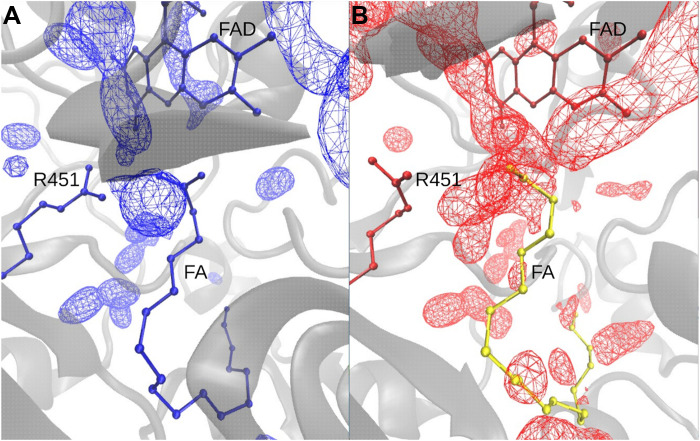
Water density maps from MD simulations. (**A**) Water density distribution (colored mesh) in the substrate-binding site and around the FAD cofactor in the presence of FAD_ox_ and bound FA. (**B**) Water density for the semiquinone form of FAD (FAD^•–^) in the absence of substrate. The experimental position of FA is shown in yellow after aligning the protein backbone for reference.

Water density maps were generated from 500-ns MD simulations to assess the solvent distribution around the active site. As shown in Fig. 10A and fig. S2, simulations of FAD in the oxidized state with two bound FAs reveal three prominent water density peaks near FAD, FA, and R451, corresponding well with the crystallographic water positions (W1 to W3) observed in the substrate-bound structure [Protein Data Bank (PDB): 6YRU]. In contrast, simulations of the reduced FAD^•–^ state without FAs (Fig. 10B and fig. S2) show five distinct water density sites near FAD, especially in the region typically occupied by FA substrates. Notably, in the reduced state (Fig. 10B), the space between the catalytic site and the pocket entrance becomes extensively hydrated, whereas in the oxidized state with two FAs (Fig. 10A), water molecules remain localized around FAD, and the binding pocket remains comparatively less hydrated and compact.

For completeness, we also performed MD simulations of the FAD_ox_ substrate–devoid system. These simulations reveal that although water molecules do accumulate near the flavin, their number and proximity are lower compared to those in the substrate-free FAD^•–^ system. Specifically, the average number of water molecules was approximately one fewer in the FAD_ox_ simulations (7.6 versus 8.7 in FAD^•–^; fig. S7). The solvent-accessible volume near the flavin was also greater in the FAD^•–^ state, with an average increase of 31.3 Å^3^ compared to the FAD_ox_ state (fig. S7). Moreover, the distance between R451 and the flavin fluctuated between values observed in the FAD_ox_/FA-bound and FAD^•−^/substrate-free systems (fig. S6). Together, these results suggest that the increased solvent accessibility observed in the FAD^•–^ state largely arises from water molecules filling the substrate-binding pocket and surrounding the reduced flavin radical.

To investigate the influence of the protein environment in *Cv*FAP on the absorption spectrum of the solvated electron, we performed preliminary simulations of the _e_^–^_H_ spectrum in *Cv*FAP, based on snapshots of the above simulations, and in bulk water (fig. S8). The simulated _e_^–^_H_ spectrum in *Cv*FAP has a broad maximum at ~700 nm, in agreement with the experimental results of Fig. 3A, and qualitatively similar to that computed for bulk water. A current limitation of this approach is that the solvated electron structures were not sampled using full quantum mechanics/molecular mechanics (QM/MM) MD but instead derived from structures obtained from MD simulations of either FAD^•−^ or neutral bulk water. These structures were then minimized using the QM/MM protocol. As a result, they may exhibit a preference for a limited number of local configurations, potentially giving rise to the multiple peaks observed in fig. S6. Nevertheless, the position of the main absorption maximum at 660 nm (1.88 eV) is in good agreement with both prior theoretical predictions and available experimental data (1.7 eV) (*26*, *27*). Within the FAP protein, we observe absorption in a similar region (~700 nm), with a slight red shift compared to bulk water. This shift may be attributed to specific electrostatic or structural effects within the protein environment, such as the vicinity of the protonated arginine R451. However, these observations are preliminary, and a detailed investigation of the origin of this red shift is beyond the scope of the present work. Further studies will be needed to fully elucidate this effect.

## DISCUSSION

Our simulations reveal the presence of a contiguous layer of water molecules close to FAD^•–^ in *Cv*FAP. At first glance, it may seem counterintuitive that the region typically occupied by the FA substrate—and subsequently the alkyl product during catalysis—could become partially filled with water molecules in their absence. However, this volume primarily encompasses the immediate vicinity of the flavin, including the site normally occupied by the substrate’s anionic carboxyl group, rather than its hydrophobic tail. This observation provides strong support for interpreting our experimental data in terms of the formation of a hydrated electron as the primary product following electron ejection from the isoalloxazine ring of *Cv*FAP. This mechanism contrasts with our previous findings in other flavoprotein oxidases, where amino acid residues were deduced to be implicated as electron acceptors (*15*).

The notable finding is that electron ejection to water under low photon energy rather than high photon energy excitation may appear surprising at first sight. However, we emphasize that two different electronically excited states are populated under these conditions. We hypothesize that electronic coupling between the D_2_ FAD^•–^ excited state and the [FAD_ox_ ArgH^•^] may allow direct flavin to arginine electron transport before D_2_ → D_1_ internal conversion, bypassing the [FAD_ox__e_^–^_H_] state populated via D_1_. It is also conceivable that the transitions display charge transfer character, allowing direct population of [FAD_ox__e_^–^_H_] and [FAD_ox_ ArgH^•^], respectively, as we have alluded to for the formation of [FAD_ox_ HisH_2_^•^] states in flavoprotein oxidases (*15*). Quantum chemical calculations of the involved potential energy surfaces may help to elucidate the origin of these intriguing properties.

On a more practical level, other excitation wavelength effects in *Cv*FAP have been reported. In particular, very recently, its overall yield was shown to be blue/violet light excitation sensitive under anaerobic conditions (where FAD^•–^ can be photoaccumulated) (*7*). It is tempting to consider the possibility that these findings are related.

Last, given that *Cv*FAP has a long substrate-binding cavity, the here-revealed photoreactions may ultimately be harnessed to photoreduce suitable long-chain molecules that can be placed close enough to the arginine receptor. If placed appropriately, then these molecules could enable forward ET to these external substrates, efficiently competing with the back electron transfer. This strategy paves the way for extending the development of substrate-specific photocatalytic reactions using engineered flavoproteins.

## MATERIALS AND METHODS

### Sample preparation

Samples of substrate-containing *Cv*FAP (substrate copurified from *Escherichia coli*; no exogenous substrates added) were prepared under red light in 10 mM tris buffer (pH 8.0) containing 150 mM NaCl, as previously described (*6*). The sample was placed in a 1-mm optical pathlength cell (Hellma model 110-QS with a shortened headspace) at a concentration corresponding to an optical density at 560 nm in the 0.3 to 0.5 range for the FAD^•–^ form. The sample was degassed as described in (*28*). Subsequently, the FAD_ox_-substrate *Cv*FAP sample was illuminated at 0°C using a 455-nm light-emitting diode (Thorlabs, M455L4) until the FAD^•–^ form was fully formed, typically within 1 min. GOX was purchased from Sigma-Aldrich, dissolved in 80 mM glycine-NaOH buffer (pH 10.1), degassed, and brought predominantly in the FAD^•–^ state by reduction with small amounts of sodium dithionite.

### Spectroscopy

Steady-state absorption spectra were recorded with a Jasco V-770 spectrophotometer. Femtosecond pump-probe spectroscopy in the visible absorption range at a repetition rate of 500 Hz was performed as described in (*28*). The ~100-fs, ~100-μm-diameter pump pulse was centered either at 390 nm (obtained by frequency doubling of the 780-nm fundamental beam) or at 560 nm (obtained using a noncollinear optical parametric amplifier). The polarization of the pump pulse was set at the magic angle (54.7°) relative to the probe pulse, and its intensity was adapted so as to excite ~10% of the sample per pulse. The cell was mounted in a Lissajous scanner, ensuring shot-by-shot sample replacement, and maintained at a temperature of 10° to 12°C.

### MD simulations

MD simulations were performed using the crystal structure available in the PDB [PDB code: 6YRU (*5*); resolution: 1.78 Å, resolved in the dark state at 100 K]. The CHARMM36m force field (*29*, *30*) was applied for protein residues, and the modified CHARMM TIP3P model (*31*) was used for water molecules. For the FAD cofactor, a specifically developed force field for flavins was used (*32*). Parameters for C_18_ FA molecules were obtained from the CHARMM General Force Field (version 2024.1) (*33*, *34*). Protonation states of titratable residues were assigned using PROPKA 3.0 (*35*), while histidine protonation states were determined by visual inspection, guided by ideal stereochemistry.

The system was then centered in a cubic box of aqueous solvent, ensuring that all protein atoms were at least 10 Å from the box edges. The final system, comprising the entire *Cv*FAP protein, FAD cofactor, C_18_ FA substrate, and solvent, contained ~27,600 water molecules. MD simulations were carried out using the GPU-accelerated NAMD program (version 3.0) (*36*, *37*). Periodic boundary conditions were applied, and long-range electrostatics were calculated using the particle mesh Ewald method (*38*). Potassium counterions were added to neutralize the system’s net charge. Van der Waals interactions were smoothly truncated using a switching function starting at 9 Å and a cutoff at 11 Å. Electrostatic interactions were evaluated every four steps, while short-range nonbonded interactions were computed at each step. A 2-fs integration time step was used with constrained bond lengths. Following energy minimization, the system was equilibrated for 100,000 steps at 295 K and 101.325 kPa, using the Berendsen thermostat and barostat (*39*) with a relaxation time of 500 fs and positional rescaling applied every four time steps. Production simulations were then conducted, and system coordinates were saved every 1 ns.

MD simulations were performed for 500 ns for *Cv*FAP in three states: (i) with oxidized flavin and two C_18_ FA (as observed in the crystal structure; PDB code: 6YRU), (ii) with oxidized flavin and one C_18_ FA in the active site, and (iii) with semireduced anionic flavin and no FA substrate. Simulations began from state (i), with both FAs present, and were run for 500 ns. Following this, the FA located farther from the flavin and catalytic site was removed, and the system was simulated for an additional 100 ns. The final structure from this simulation was then used to initiate state (iii), in which the remaining FA in the catalytic site was removed and the flavin parameters were updated to represent the semireduced anionic form (*32*). For each state, two independent simulations were performed using different initial random velocities to ensure statistical convergence.

Density maps were generated using the VolMap tool in VMD 1.9.4 (*40*), based on all trajectory frames and a grid resolution of 0.4 Å. The RDF of water molecules was calculated using the CHARMM program (*41*) using a grid spacing of 0.2 Å and relative to the N_5_ atom of the flavin, to analyze the number of water molecules within concentric shells as a function of distance. Solvent-accessible volume around the N_5_ atom was analyzed using the coor search command in CHARMM (*41*).

### Spectral calculations in FAP and vacuum

Electronic absorption spectra of neutral radical forms of arginines R451 and R145, as well as cysteine C432, within the FAP protein were calculated using a hybrid QM/MM approach (*42*). The FAP system was divided into QM and MM regions, with the QM region comprising the side chain atoms of the target residue—assumed to act as the electron acceptor—up to the Cγ atom for arginine or Cβ for tyrosine. The bond between the Cβ (Cγ) and Cα (Cβ) atoms was capped with a link hydrogen atom. An additive QM/MM coupling scheme with electrostatic embedding was used, as implemented in the pDynamo software suite (*43*, *44*).

The MM region, comprising ~91,500 atoms, was described using the CHARMM force field as detailed in the “MD simulations” section above. The QM region was treated using density functional theory (DFT) with the def2-TZVP basis set (*45*), which includes polarization functions on all atoms. All QM(DFT)/MM calculations used the RIJCOSX algorithm (*46*) for efficient evaluation of two-electron integrals, where the coulomb term is approximated using the resolution of identity method, and the exchange term is treated via seminumerical integration. In addition, D3 dispersion corrections with Becke-Johnson damping were included (*47*). Geometry optimizations and electronic spectral calculations were carried out using the QM(DFT)/MM potential in pDynamo (*43*, *44*) interfaced with the ORCA quantum chemistry program (*48*). No cutoffs were applied for nonbonded interactions.

Spectral calculations were performed on 100 structures extracted at 1-ns intervals from a 500-ns MD trajectory of the FAP system containing the anionic flavin radical (FAD^•−^). Before spectral calculations, each structure was geometry optimized using the QM/MM approach described above. To model the state immediately following ET from FAD, the cofactor was represented using force field parameters corresponding to the oxidized flavin (*32*). For each optimized structure, vertical excitation energies and oscillator strengths of the lowest 50 excited states were computed using time-dependent DFT (TD-DFT) (*49*) with the unrestricted B3LYP functional (*50*). The final absorption spectrum was obtained by averaging the contributions from all transitions across the 100 structures. Oscillator strengths from all structures were combined and convoluted using a Gaussian line shape with a peak width of 25 nm, as implemented in the Avogadro software (*51*).

To calculate absorption spectra of the solvated electron, the QM region included all water molecules within 7 Å of both the N_5_ atom of FAD and the side chain of residue R451. Across the ensemble of 100 MD snapshots, this region typically comprised an average of eight water molecules per frame. For TD-DFT calculations involving the solvated (hydrated) electron, the CAM-B3LYP functional was used in conjunction with the augmented correlation-consistent triple-zeta basis set (aug-cc-pVTZ). This combination of a long-range corrected functional and a diffuse basis set was included to accurately describe the highly diffuse nature of the excess electron (*27*). Before the calculation of absorption spectra, each structure was geometry optimized at the B3LYP/aug-cc-pVTZ level of theory, with all atoms located more than 15 Å from the QM region constrained to their initial positions.

For comparison, spectral simulations in vacuum were carried out for model compounds representing the side chains of arginine and cysteine (up to the Cβ atom), using the same DFT/TDDFT methodology as described above, except that oscillator strengths from all structures were combined and convoluted using a Gaussian line shape with a peak width of 25 nm.
