# Supplementary material for: Ultrafast photooxidation of semireduced flavin in fatty acid photodecarboxylase
Source: Sci Adv. 2025 Sep 19;11(38):eadz1904. doi: 10.1126/sciadv.adz1904 (PMC12448124; doi:10.1126/sciadv.adz1904)
Supplement: Supplementary file 1 — Figs. S1 to S8 [file sciadv.adz1904_sm.pdf]

Science Advances
AAAS

Supplementary Materials for

Ultrafast photooxidation of semireduced flavin in fatty acid photodecarboxylase

Marten H. Vos *et al.*

Corresponding author: Marten H. Vos, marten.vos@polytechnique.edu;
Alexey Aleksandrov, alexey.aleksandrov@polytechnique.edu

Sci. Adv. **11**, eadz1904 (2025)
DOI: 10.1126/sciadv.adz1904

This PDF file includes:

Figs. S1 to S8

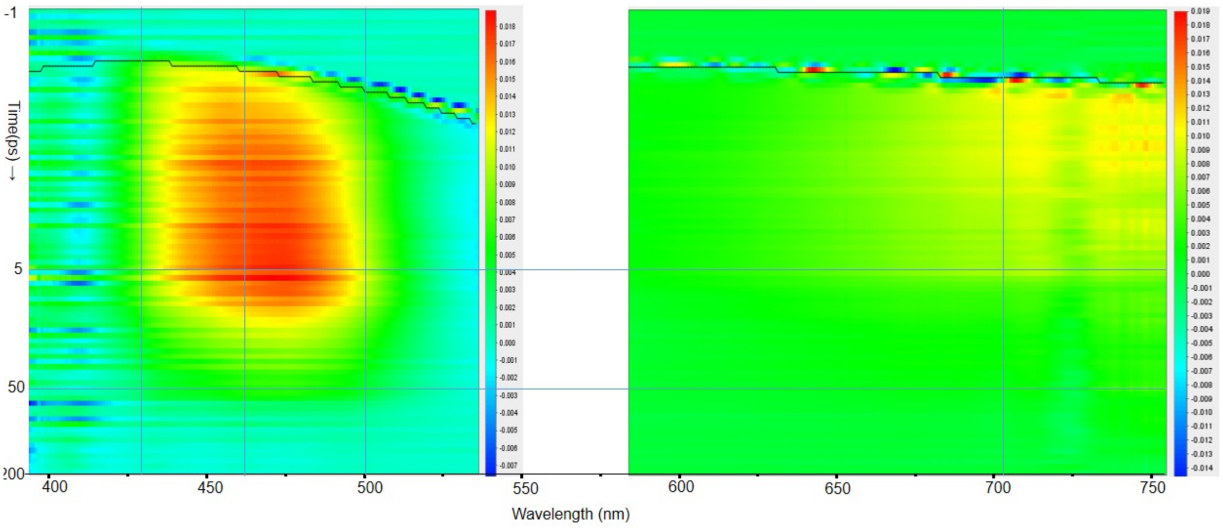

Fig. S1. Two-dimensional false color transient absorption map of FAD$^{\bullet-}$ in *Cv*FAP under excitation at 560 nm. The fitted temporal dispersion is shown as a solid line. The horizontal lines correspond to changes in time windows. The vertical lines correspond to kinetics shown in Fig. 2A of the main text. The two time windows derive from separate measurements covering different spectral regions and temporal dispersions, but were recorded under identical excitation conditions.

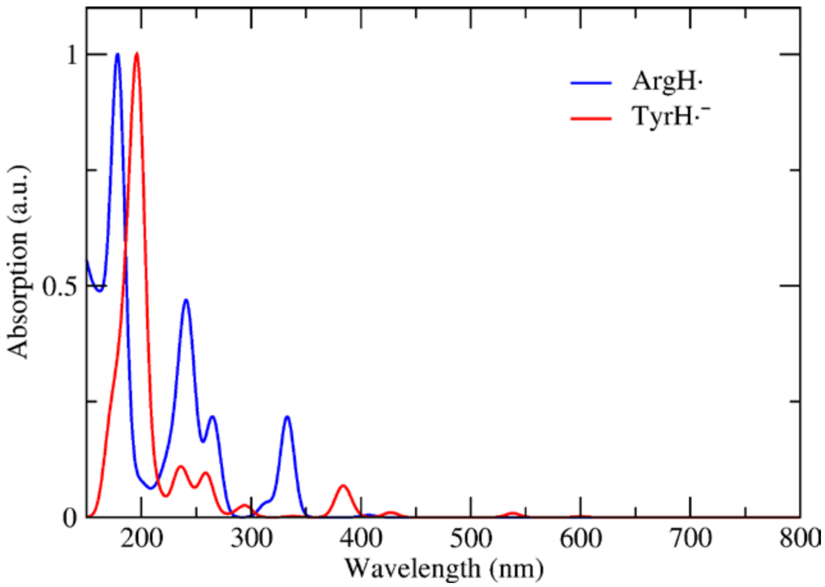

Fig. S2. Simulated absorption spectra of model compounds representing arginine and tyrosine radicals. Spectra for ethylguanidine (arginine side chain) and 4-methylphenol (p-cresol; tyrosine side chain) were calculated in the gas phase.

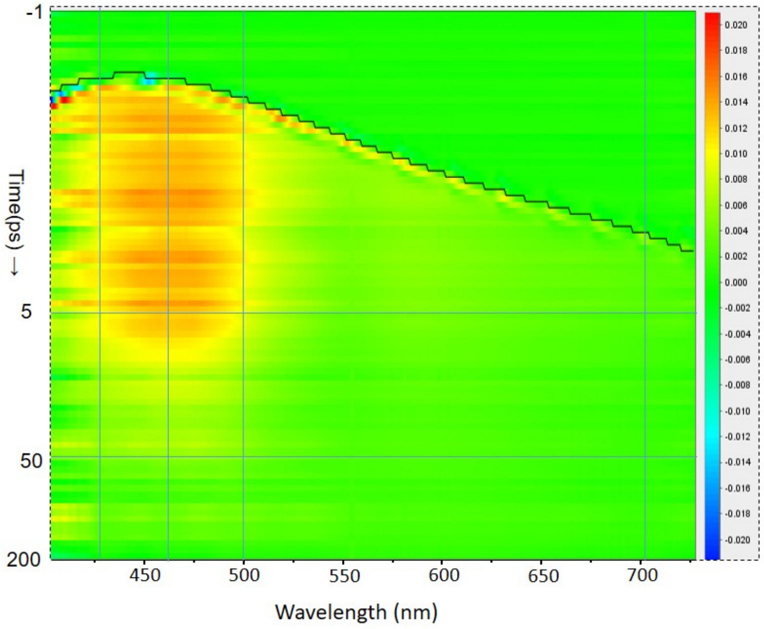

Fig. S3. Two-dimensional false-color transient absorption map of FAD$^{\bullet-}$ in *Cv*FAP following excitation at 390 nm. The solid line indicates the fitted temporal dispersion. Horizontal lines mark transitions between different time windows. Vertical lines denote probe wavelengths corresponding to the kinetic traces shown in Fig. 2B of the main text.

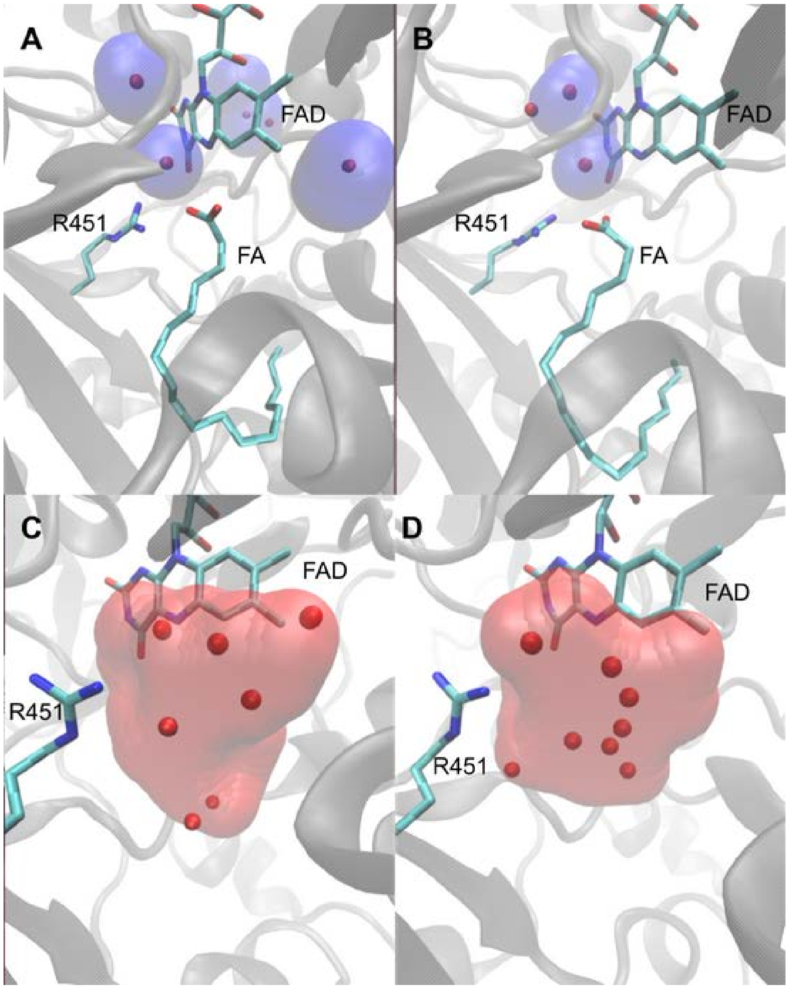

Fig. S4. Water density maps derived from MD simulations. Panels A and B: Water density within 10 Å of the isoalloxazine ring for FAD_{ox} bound to a fatty acid (FA), shown for two independent MD simulations. Panels C and D: $FAD^{\bullet -}$ without any substrate, also from two independent simulations. Water molecules within 5 Å of FAD non-hydrogen atoms are shown as spheres for a representative snapshot selected to reflect the average number of nearby water molecules.

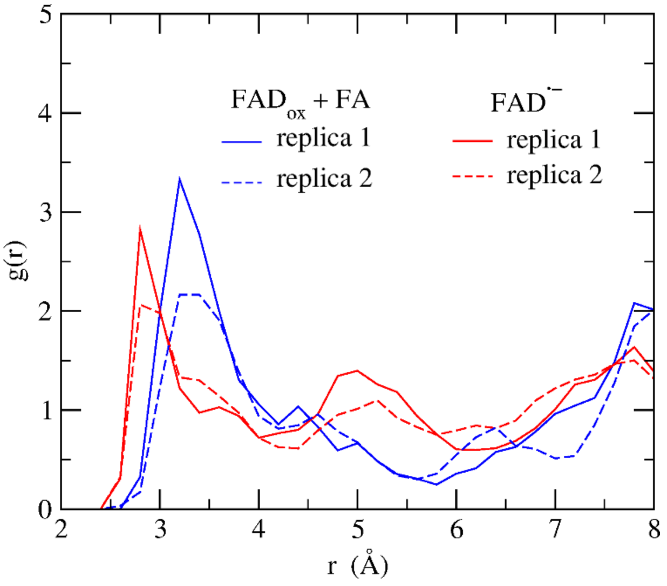

Fig. S5. Radial distribution function (RDF) analysis of water around the N5 atom of FAD. The RDF reveals a higher probability of finding water molecules near the N5 atom in the reduced state without fatty acids (~2.8 Å) compared to the oxidized state without fatty acids (~3.5 Å). Results from two replicas per system are shown.

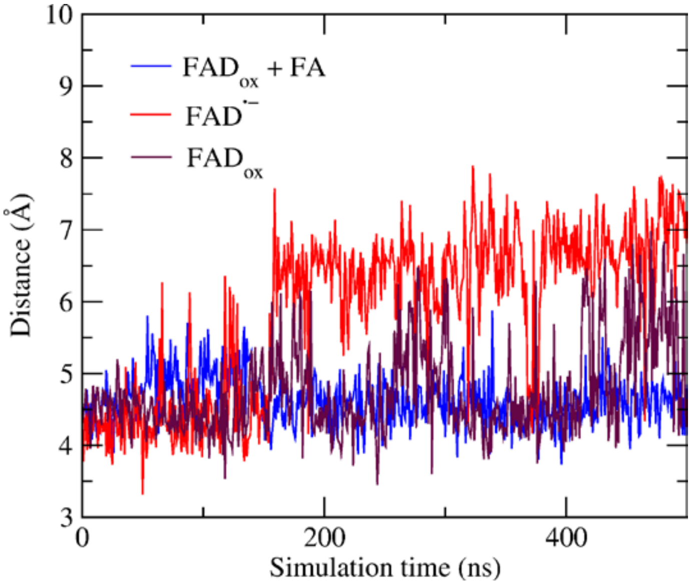

Fig. S6. Time evolution of the distance between the isoalloxazine ring of FAD and the guanidinium group of R451 during MD simulations. Shown are trajectories for three systems: FAD_{ox} with bound fatty acid, FAD_{ox} without any substrate or product, and reduced $FAD^{•-}$ without FA.

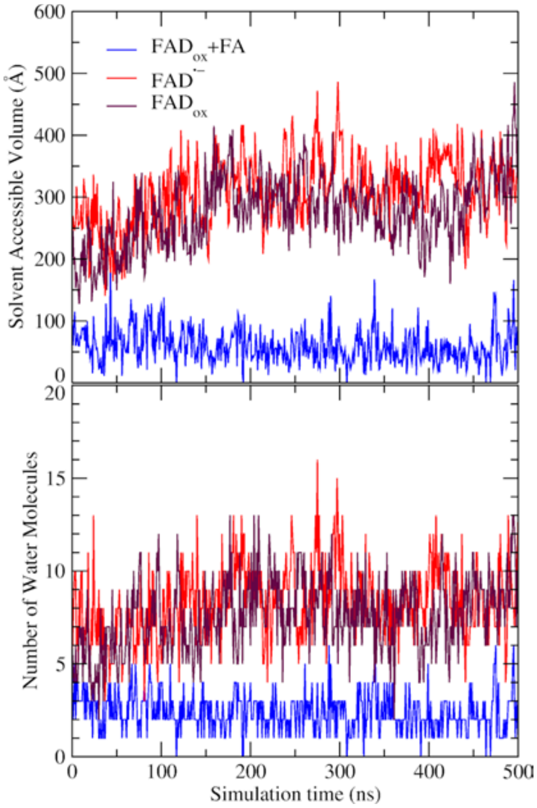

Fig. S7. Water molecules near FAD. *Top panel:* Solvent-accessible volume of the pocket surrounding the N5 atom of FAD over the course of molecular dynamics simulations. *Bottom panel:* Number of water molecules within the solvent-accessible region near the isoalloxazine ring of FAD. Data are shown for three systems: FAD_{ox} with bound FA, FAD_{ox} without any substrate or product, and reduced $FAD^{\bullet-}$ in the absence of FA.

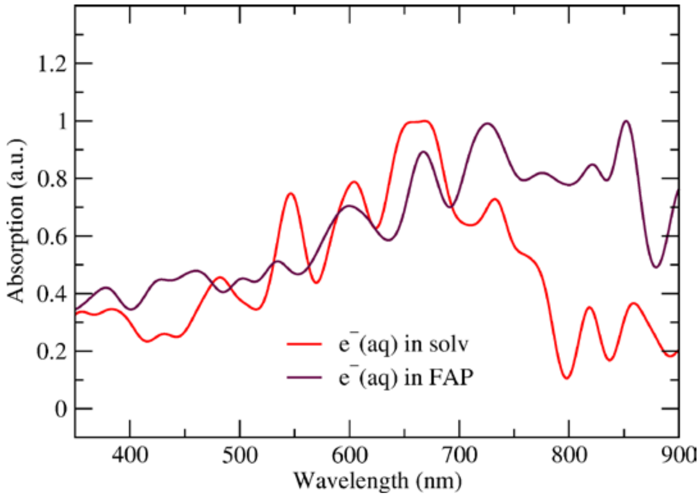

Figure S8. Simulated absorption spectra of the solvated electron in FAP and in solvent. The spectra were averaged over 100 frames extracted from MD simulations.
